# A multi-species direct-fed microbial supplement alters the milk lipidome of dairy cows

**DOI:** 10.3168/jdsc.2022-0244

**Published:** 2022-11-18

**Authors:** Adeoye O. Oyebade, Godstime A. Taiwo, Modoluwamu Idowu, Taylor Sidney, Diwakar Vyas, Ibukun M. Ogunade

**Affiliations:** 1 Department of Animal Sciences, Institute of Food and Agricultural Sciences, University of Florida, Gainesville 32611; 2 Division of Animal and Nutritional Sciences, West Virginia University, Morgantown 36506

## Abstract

The study evaluated the effects of supplementing a multi-species direct-fed microbial (DFM) on the milk lipidome of lactating dairy cows. Twenty-four multiparous Holstein cows (41 ± 7 d in milk) were used in a randomized complete block design with experimental duration of 91 d. Cows were blocked based on energy-corrected milk yield from a 14-d pretreatment period, and were assigned randomly within each block to the following treatments: (1) control (CON): corn silage-based total mixed ration without DFM; or (2) BOV+: basal diet top-dressed with a DFM containing a mixture of *Lactobacillus animalis* (LA-51), *Propionibacterium freudenreichii* (PF-24), *Bacillus subtilis* (CH201), and *Bacillus licheniformis* (CH200) at 11.8 × 10^9^ cfu/d. Milk samples were taken from morning and evening milkings on 2 consecutive days of each week of the pretreatment and treatment periods. Separate composites of pretreatment period and treatment period samples were prepared for individual cows and used for lipidome analysis. Lipidome analysis of the milk samples was performed using an ultra-high-performance liquid chromatograph linked to a quadrupole time-of-flight mass spectrometer in both positive and negative ionizations. The relative concentrations of 14 lipid species, including long-chain polyunsaturated fatty acids (LC-PUFA) such as FA 20:8 and FA 28:7 and triacylglycerides (TG) such as TG 40:3 and TG 54:2, were increased [false discovery rate (FDR) ≤0.05], whereas 13 lipid species, including saturated FA 24:0 and TG 40:0 were decreased (FDR ≤0.05) by supplemental BOV+. The relative concentration of de novo FA in milk was greater, whereas that of preformed FA was lower in dairy cows supplemented with BOV+. Results from this study demonstrate the potential of a DFM containing *L. animalis, P. freudenreichii, Bacillus subtilis*, and *B. licheniformis* to alter the milk lipidome in lactating dairy cows toward increased relative concentration of LC-PUFA, which might offer a healthier profile of FA to consumers with its associated health benefits.

## Highlights

• Dairy cows were fed typical lactating diets with or without direct-fed microbial.
• Lipidome analysis of milk samples from the cows was performed.
• The direct-fed microbial altered the milk lipidome of the dairy cows.

## Graphical Abstract

**Figure:**
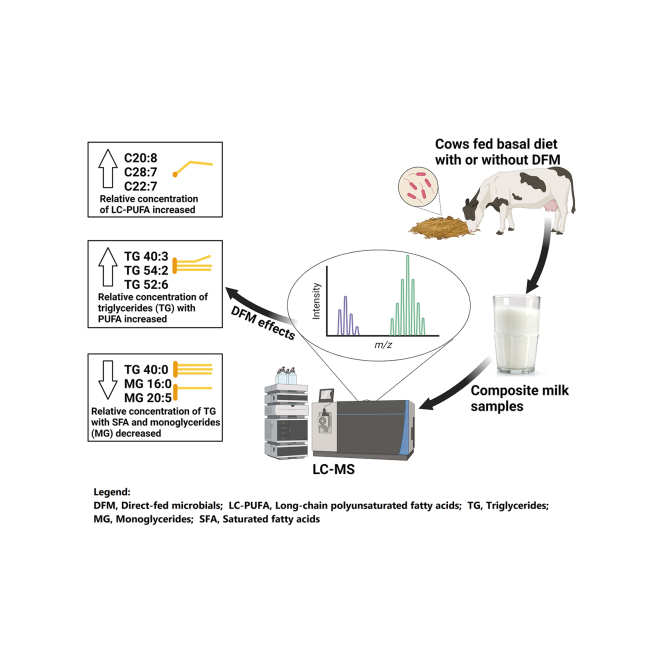
**Summary:** We determined the effect of a direct-fed microbial supplement containing a mixture of *Lactobacillus animalis, Propionibacterium freudenreichii, Bacillus subtilis*, and *Bacillus licheniformis* on the milk lipidome of dairy cows in early lactation. Our results showed that the supplemental additive altered the milk lipidome toward increased relative concentrations of polyunsaturated fatty acids (PUFA), which would increase PUFA consumption in place of saturated fatty acid, resulting in potential health benefits such as reduced risk of coronary heart disease in consumers of milk.

Direct-fed microbials (**DFM**) are commonly fed to improve the performance and health of dairy cows (McAllister et al., 2011); however, no studies have attempted to evaluate the effects of DFM supplementation on the nutritional value of milk, beyond milk components such as total milk total protein and fat. In recent years, interest in the nutritional value and health benefits of food products of animal origin has been growing (Henchion et al., 2017). Fat is one of the most important components in bovine milk, and its concentration ranges from 3 to 6%, depending on several factors including breed, diet, stage of lactation, and season (Linn, 1988; Palmquist et al., 1993). The fats in bovine milk are highly complex and contain thousands of lipid species with different health benefits (Sichien et al., 2009); thus, comprehensive analysis of milk lipid species via lipidomics analysis will provide more information related to health benefits and nutritive quality of milk fat. The field of lipidomics, which is based on analytical tools, especially high-resolution MS, has enabled comprehensive analysis of lipid molecular species, including their quantitation and metabolic pathways in different types of samples including milk, blood, and meat (Yang and Han, 2016; Xu et al., 2020).

In our previous study (Oyebade et al., 2022, unpublished), dietary supplementation of a mixture of *Lactobacillus animalis* (LA-51), *Propionibacterium freudenreichii* (PF-24), *Bacillus subtilis* (CH201), and *Bacillus licheniformis* (CH200) increased ether extract digestibility and yields of milk fat and FCM. Increased milk fat yield as a result of increased dietary fat digestibility and uptake by the mammary gland is thought to alter milk fatty acid composition (Palmquist and Jenkins, 1980). Therefore, we hypothesized that BOV+ supplementation would alter the milk lipidome of dairy cows. The objective of this study was to determine whether dietary supplementation of a mixture of *L. animalis, P. freudenreichii, B. subtilis*, and *B. licheniformis* would alter the milk lipidome of early-lactation dairy cows.

All animal care and experimental procedures for this study were approved by the University of Florida Institutional Animal Care and Use Committee (Protocol number: 201810520).

Twenty-four lactating multiparous Holstein cows in early lactation (mean ± SD: 41 ± 7 DIM) with an average daily milk yield of 43 ± 7 kg/d and average BW of 677 ± 64 kg were used in the study. The dairy cows were assigned to 12 blocks based on pretreatment ECM yield. Within each block, the cows were randomly assigned to 1 of 2 dietary treatment groups: (1) a corn silage-based diet with no additive (control, **CON**; n = 12), and (2) the basal diet top-dressed with a mixture of *L. animalis, P. freudenreichii, B. subtilis*, and *B. licheniformis* at 11.8 × 10^9^ cfu/day (Bovamine Dairy Plus: **BOV+**; n = 12). The cows were housed in a freestall, open-sided, sand-bedded barn fitted with Calan gates (American Calan) for individual feeding, with 2 rows of fans and misters with low-pressure nozzles for cooling the cows. The composition of BOV+ was based on the expected interaction between *L. acidophilus* and *P. freudenreichii* to sustain lactate production and increase overall productivity, coupled with an additive effect from *Bacillus* spp., which, like *L. acidophilus* and *P. freudenreichii*, have been shown to improve milk composition (Boyd et al., 2011; West and Bernard, 2011; Sun et al., 2013). Supplemental BOV+ was mixed with dried molasses just before supplementing as a top-dress; control cows received only molasses as a top-dress.

The cows were fed the treatment diets for 91 d, after a 14-d pretreatment (covariate) period during which all cows were fed the same basal diet. The basal diet was fed as a TMR composed of 47.1% corn silage, 18.8% corn grain, 15.1% soybean meal, 4.7% citrus pulp, 7.4% whole cottonseed, 1.7% Palmit-80 (Global Agri Trade Corporation), and 5.2% mineral and vitamin mix. This diet was formulated to meet the energy and protein requirements of lactating dairy cows producing at least 42 kg/d of milk yield, with 3.50% milk fat and 3.20% milk protein. The basal diet was formulated using NDS Professional (RUM&N, which is based on Cornell Net Carbohydrate and Protein System equations (CNCPS v. 6.5). Cows were fed twice daily, at 0600 and 1200 h, 60% and 40% of the total daily allotment, respectively. Feed offered was adjusted daily based on preceding 3 days' average intake and fed ad libitum for minimum daily refusals of 5 to 10%. The cows were milked twice daily at 1000 and 2200 h. Milk samples were collected from morning and evening milkings twice a week during the study, starting from the pretreatment period (d −14 to 0) and experimental period (d 8–91). All milk samples from the morning and evening milkings for all cows were immediately stored at −20°C. At the end of the study, milk samples were thawed, and equal aliquots were taken from all morning and evening milkings and composited on an individual cow basis separately for the pretreatment and treatment periods (pretreatment period; n = 12 for each dietary group, and treatment period; n = 12 for each dietary group; making a total of 24 samples for each of the pretreatment and treatment periods). The samples were composited per cow for each of the periods because the major interest of this study was to evaluate the average effect of DFM on the milk lipidome, rather than changes in milk lipid composition over time.

Lipid extraction from all composited milk samples was done using a modified Folch liquid-liquid extraction protocol with dichloromethane and methanol (Folch et al., 1957; Zardini Buzatto et al., 2021). A pooled mixture of all the milk samples was prepared using equal aliquots of all samples and was used as the quality control (**QC**) sample. The detailed sample extraction procedure and analysis has been recently published (Zardini Buzatto et al., 2021).

Lipidome analysis of the extracted milk samples was performed using a Thermo Vanquish ultra-high-performance liquid chromatograph (Thermo Fisher Scientific) linked to Bruker Impact II quadrupole time-of-flight MS (Bruker Daltonics) in both positive and negative ionizations. Each randomized batch of 8 samples was injected in between 2 injection replicates of the QC aliquot extracted with that batch. All 24 samples each from the pretreatment and treatment periods (total of 48 samples) were injected in duplicates, for a total of 96 sample injections that were performed in each ionization polarity. Tandem MS (MS/MS) spectra were acquired for all samples for identification. The liquid chromatography-MS data from the 96 sample injections (as well as QC injections) were independently processed in positive and negative ionizations. The data acquired in positive and negative ionization from each sample extraction were combined; that is, the detected features from all samples were merged into one feature-intensity table.

A 3-tier identification approach based on MS/MS identification and accurate mass match was used for lipid identification (Zardini Buzatto et al., 2021). The parameters used for identification were MS/MS match score ≥500; precursor *m*/*z* error ≤5.0 mDa for tier 1, MS/MS match score ≥100; precursor *m*/*z* error ≤5.0 mDa for tier 2, and mass match with *m*/*z* error ≤5.0 mDa and 20 ppm for tier 3. After tier 3 identification, a 6-tier filtering and scoring approach was used to restrict the number of matches and select the best identification option to determine the lipid sub-classes for normalization (Zardini Buzatto et al., 2020). All compounds identified in tiers 1, 2, and 3 were combined for normalization and statistical analysis.

Data normalization was performed by using Lipidomix (Splash Lipidomix Mass Spec Standard, Avanti Polar Lipids), a quantitative standard mixture of deuterated lipids of various lipid classes (Drotleff et al., 2020). The positively and putatively identified lipids were matched to 1 of the 14 internal standards according to lipid class similarity and expected retention time range for each class. Intensity ratios (intensity of each lipid divided by intensity of the matched internal standard) were calculated for normalization (Zardini Buzatto et al., 2021).

Statistical analysis was performed with MetaboAnalyst 5.0 (https://www.metaboanalyst.ca/). Noninformative features [internal standards and features with near-constant values between the groups determined by low relative standard deviation (RSD)] and features with low repeatability (RSD >30% for QC samples) were filtered out (Zardini Buzatto et al., 2021). The data set was further normalized by auto-scaling and log-transformation. Finally, the auto-scaled intensity ratios were used for multivariate and univariate analyses. A partial least squares discriminant analysis (**PLS-DA**) score plot was generated to visualize the difference between the 2 dietary groups. Univariate analysis (volcano plot) was used to determine the differentially abundant lipid species using a false discovery rate (**FDR**) ≤0.05. Treatment effects on the relative concentrations of preformed free fatty acids (**FA**; with >16 carbons) and de novo FA (FA with 4–14 carbons) in milk were determined at *P* ≤ 0.05.

During the pretreatment period, a total of 9 lipid species [FA 84:0, triacylglyceride (**TG**) 80:11, TG 82:12, cardiolipin 18:1, FA 12:2, FA 14:1, FA 18:3, FA 21:1, and FA 20:1] showed a tendency to be differentially abundant (FDR ≤0.10) between the 2 dietary groups, which was likely due to animal-to-animal variation. Therefore, subsequent paragraphs describe the results of lipidome data obtained during the treatment period, excluding those that were differentially abundant during the pretreatment period.

A total of 7,143 lipid species were detected and identified (https://doi.org/10.13140/RG.2.2.17897.57444). The most abundant lipid species detected in all milk samples were TG, followed by diglycerides (**DG**), sterol lipids, and FA. The PLS-DA scores plot showed a clear separation between the 2 groups (Figure 1), indicating that BOV+ altered the milk lipidome of the dairy cows. The PLS-DA permutation test (empirical *P*-value of 0.015 for 1,000 permutations) confirmed the validity of the separation between the 2 groups. The result of the volcano plot analysis showed that, relative to CON, the relative concentrations of 14 lipid species were increased (FDR ≤0.05) and 13 lipid species were decreased (FDR ≤0.05) by supplemental BOV+ (Table 1). All differentially abundant lipid species belonged to several lipid classes with long carbon chains, such as FA, TG, DG, monoacylglycerides (**MG**), sphingolipids, glycerophospholipids, and fatty acid estolides (Table 1).

**Figure 1:**
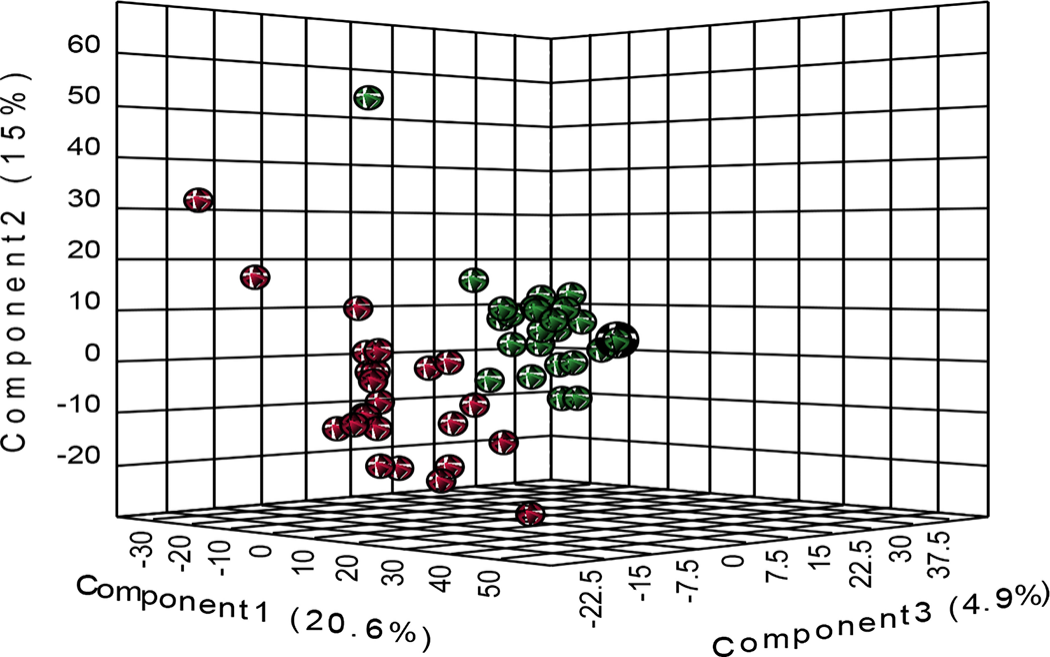
Partial least squares discriminant analysis scores plot of the milk lipidome of dairy cows fed a diet supplemented a direct-fed microbial, consisting of a mixture of *Lactobacillus animalis, Propionibacterium freudenreichii, Bacillus subtilis*, and *Bacillus licheniformis* (BOV+; red symbols) versus control cows (green symbols).

**Table 1:** Effects of multi-species direct-fed microbial (BOV+1) on milk lipidome of dairy cows^2^

Lipid class/subclass	Name	FC	FDR
Free fatty acids	FA 20:8	1.53	0.05
Free fatty acids	FA 28:7	1.53	0.03
Free fatty acids	FA 22:7	1.38	0.01
Free fatty acids	FA 24:0	0.45	0.01
Triacylglycerols	TG 40:3	2.25	0.01
Triacylglycerols	TG 54:2	1.37	0.04
Triacylglycerols	TG 52:6	1.36	0.00
Triacylglycerols	TG 48:4	1.33	0.03
Triacylglycerols	TG 42:3	1.26	0.05
Triacylglycerols	TG 40:0	0.49	0.01
Monoacylglycerols	MG 16:0	0.53	0.03
Monoacylglycerols	MG 20:5	0.43	0.05
Diacylglycerols	DG 32:2	0.66	0.04
Sphingolipids			
Sphingomyelins	SM 38:2	1.55	0.04
Ceramides	Cer 53:3	1.53	0.03
Hexosyl ceramides	HexCer 41:0	1.47	0.03
Hexosyl ceramides	HexCer 37:2	1.45	0.02
Hexosyl ceramides	HexCer 31:6	1.38	0.05
Ceramides	Cer 35:0	0.72	0.04
Ceramides	ACer 43:0	0.65	0.00
Glycerophospholipids			
Phosphoinositol monophosphates	PIP2 68:0	0.52	0.03
Diacylglycerophosphoserines	PS 39:2	0.61	0.03
Diacylglycerophosphoethanolamines	PE 28:8	0.64	0.03
Diacylglycerophosphoethanolamines	PE 18:2	0.59	0.01
Diacylglycerophosphoethanolamines	PE 25:1	0.58	0.03
Sterol lipids	ST 29:4	1.95	0.01
Fatty acid estolides	FAHFA 40:9	0.63	0.01

1 BOV+ = a mixture of *Lactobacillus animalis, Propionibacterium freudenreichii, Bacillus subtilis*, and *Bacillus licheniformis.*

2 FC = fold change of BOV+ relative to unsupplemented control; FDR = false discovery rate-adjusted *P*-value. Only lipid species with both FC ≥1.2 or ≤0.83 (relative to control) and FDR ≤0.05 are shown.

Of the free fatty acids that were altered, the relative concentrations of 3 long-chain polyunsaturated fatty acids (**LC-PUFA**): FA 20:8, FA 28:7, and FA 22:7 were increased, whereas 1 long-chain saturated fatty acid (**LC-SFA**; FA 24:0) was lower in the milk of dairy cows fed supplemental BOV+ relative to CON. The relative concentration of de novo FA in milk was greater (*P* = 0.02) and that of preformed FA was lower (*P* = 0.04) in dairy cows fed supplemental BOV+ (Figure 2).

**Figure 2:**
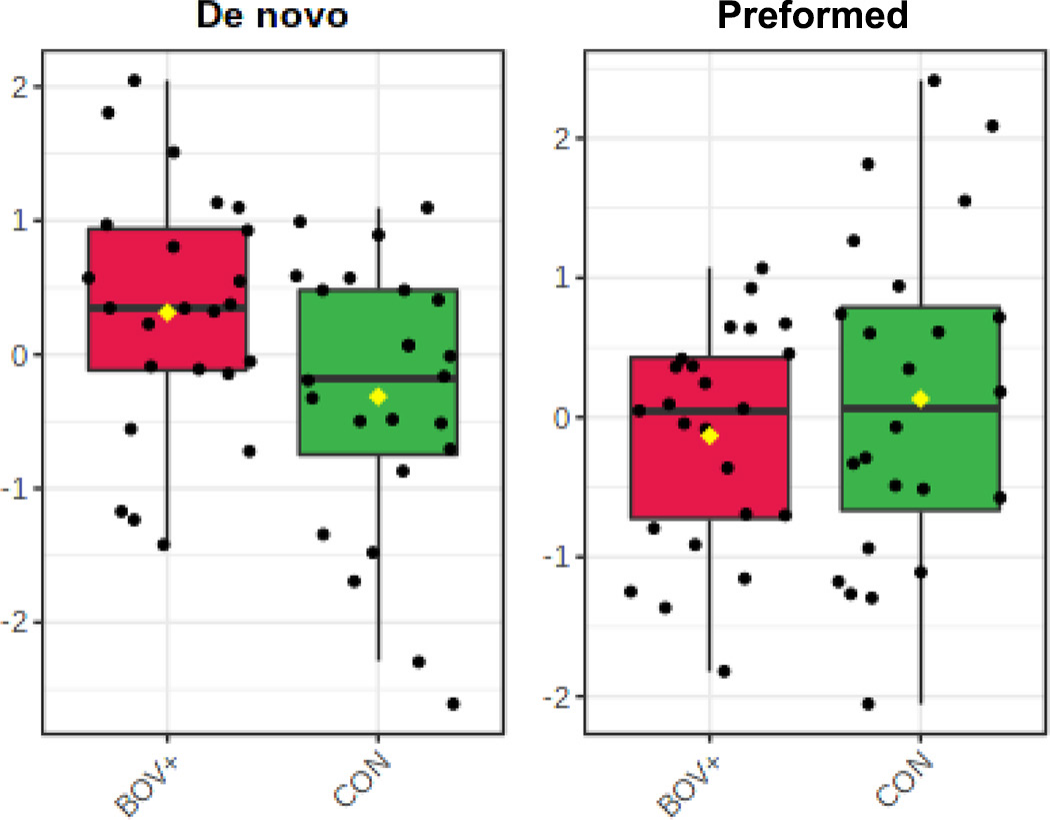
Comparison of the relative concentrations of milk de novo and preformed fatty acids in dairy cows fed a diet supplemented with a direct-fed microbial, consisting of a mixture of *Lactobacillus animalis, Propionibacterium freudenreichii, Bacillus subtilis*, and *Bacillus licheniformis.* De novo FA (FA with 4–14-carbon chain length): *P*-value = 0.02; preformed FA (FA with >16-carbon chain length): *P*-value = 0.04. CON = control (basal diet without supplementation); BOV+ = diet with direct-fed microbial. Boxplot: the box shows the interquartile range, the horizontal line within the box shows median values, diamond symbols show mean values, and the whiskers/vertical lines show maximum (top) and minimum (bottom) values.

Five types of TG with polyunsaturated bonds (TG 40:3, TG 54:2, TG 52:6, TG 48:4, TG 42:3) were increased by supplemental BOV+ relative to CON. In contrast, 1 TG with saturated LC bond (TG 40:0), 2 MG (MG 20:5; MG 16:0), and 1 DG (DG 32:2) were reduced by BOV+ relative to CON. Five types of sphingolipids (SM 38:2, Cer 53:3, HexCer 41:0, HexCer 31:6, and HexCer 31:6) were increased by supplemental BOV+. In contrast, 2 types of sphingolipids, both with saturated bonds (Cer 35:0 and ACer 43:0), were reduced relative to CON. Five types of glycerophospholipids (PIP2 68:0, PS 39:2, PE 28:8, PE 18:2, PE 25:1) and 1 type of fatty acid estolide (FAHFA 40:9) were reduced by supplemental BOV+ relative to CON.

In ruminants, long-chain FA (**LCFA**) are derived mainly from the feed and are extensively biohydrogenated by rumen microorganisms; thus, a large proportion of fats leaving the rumen are saturated (Bionaz et al., 2020). Lipid metabolism, including de-esterification and biohydrogenation, and the extent to which they occur by the actions of ruminal microorganisms have a significant influence on LCFA profiles of animal products, such as milk and meat (Demeyer and Doreau, 1999). Escape of unprotected UFA from biohydrogenation depends primarily on microbial factors that influence rate of lipolysis and biohydrogenation (Jenkins, 1993). As such, BOV+ supplementation may have contributed to the altered milk lipidome toward increased relative concentrations of LC-PUFA observed in this study. This effect might be at the rumen level, such as alteration of lipid metabolism to increase duodenal flow of UFA, which are then available for incorporation into milk fat. Alternatively, the effect might be a more complex postruminal biological effect on the host animal itself, such as effects of DFM that might influence postabsorptive lipid metabolism, composition of lipids reaching the mammary gland, and de novo FA synthesis in the mammary gland. Previous studies have demonstrated that certain strains of *B. subtilis* and *B. licheniformis* possess an acyl-lipid desaturase, an iron-dependent integral membrane protein able to selectively introduce *cis*-double bonds into LCFA; consequently, these microbes can synthesize UFA species of different lengths and branching patterns (Fulco, 1969; Diaz et al., 2002; Altabe et al., 2003). Thus, it is reasonable to speculate that dietary supplementation of BOV+ may have increased the desaturation of rumen lipid contents, which may have led to increased duodenal flow of LC-PUFA that are directly absorbed in the small intestine. Another explanation for increased duodenal flow of PUFA is the reduced biohydrogenation of dietary PUFA, which might have been due to changes in the population of rumen microbes responsible for biohydrogenation, which likewise could be due to effects of other microbes or ruminal conditions, such as rumen pH. Pattnaik et al. (2001) reported that a bacteriocin from *B. licheniformis*, lichenin, shows activity against several strains of *Butyrivibrio*, which are a major contributor to UFA biohydrogenation in the rumen. Given the role of *Bacillus* spp. in producing exogenous lipases (Gupta et al., 2004; Bracco et al., 2020), lipolysis and antagonistic actions toward microbes contributing to biohydrogenation might have occurred concomitantly. Earlier studies have shown that free FA are more digestible than esterified fats, just as UFA are more digestible than SFA; therefore, such a combination of events could increase overall absorption of UFA in the lower gastrointestinal tract, which could be responsible for the alteration of milk lipidome observed in the current study (Elliott et al., 1999; Daley et al., 2018). Because major contributors to biohydrogenation such as *Butyrivibrio* are pH sensitive, another possible explanation for increased milk concentrations of LC-PUFA is low rumen pH. Several studies have indicated that rumen pH <6.0 can reduce the extent of rumen lipolysis and biohydrogenation, which is expected to increase duodenal flow of LC-PUFA (Doreau et al., 1997; Dewanckele et al., 2017); however, we observed no effects of supplemental BOV+ on rumen pH in our other study (Oyebade et al., 2022, unpublished). Other studies evaluating rumen pH when *L. animalis* and *P. freudenreichii* were fed to dairy cows alone or in combination did not find a negative effect of the strains on rumen fermentation and pH (Raeth-Knight et al., 2007). Similarly, studies feeding a combination of *B. subtilis* and *B. licheniformis* to multiparous cows demonstrated that the microbial additive had no effect on rumen pH but increased the concentrations of milk branched-chain fatty acids, which are commonly synthesized by bacilli and used as part of their cell wall (Lamontagne et al., 2019). Further studies are needed to better understand how dietary supplementation of a mixture of *L. animalis, P. freudenreichii, B. subtilis*, and *B. licheniformis* alters rumen fermentation, with a focus on its effects on ruminal lipid metabolism and concentrations of lipid species.

High levels of UFA are toxic to several rumen microbes, especially fibrolytic bacteria (Jenkins, 1993); thus, high ruminal concentrations of UFA are expected to reduce rumen microbial fermentation and fiber digestibility. Nevertheless, in our other study, supplemental BOV+ did not affect fiber digestibility but increased FCM yield (Oyebade et al., 2022, unpublished). Published articles looking at the effect of *Lactobacillus* spp. and *P. freudenreichii* reported no or positive effect on NDF digestibility when these bacteria are fed to dairy cows (Raeth-Knight, 2007; Boyd et al., 2011). Specific *Bacillus* strains are known for their capacity to synthesize digestive enzymes, including fibrolytic enzymes such as cellulase and xylanase (Oliveira et al., 2016). Several studies have demonstrated that the combination of *B. subtilis* and *B. licheniformis* has the capacity to increase fiber digestibility of a variety of fiber sources, including grasses and legumes (Qiao et al., 2010; Oliveira et al., 2016). Indeed, in the current study, dairy cows fed supplemental BOV+ had greater relative concentrations of milk de novo FA, which is often used to indicate conditions of rumen fermentation (Woolpert et al., 2016). This is because de novo FA in milk are primarily synthesized in the mammary gland using acetate and butyrate, which are derived primarily from rumen fermentation of fibrous feeds (Palmquist et al., 1993).

Previous studies have demonstrated that apparent digestibility of fat containing unsaturated long-chain FA in the intestine is greater than that of SFA because saturated TG are more resistant to intestinal lipolysis than unsaturated TG (Elliott et al., 1999; Daley et al., 2018). In fact, total fat digestibility has been reported to increase as more UFA reach the duodenum (Boerman et al., 2015; Daley et al., 2018). This may explain the increased total-tract fat digestibility observed in dairy cows fed supplemental BOV+ in our other study (Oyebade et al., 2022, unpublished).

Polyunsaturated FA in milk and other dairy products are known to improve the health status of consumers, with health-promoting effects such as anticarcinogenic and antiatherosclerotic effects (Jensen, 2002; Micha and Mozaffarian, 2010). Mozaffarian et al. (2010) showed, in their systematic review, that a shift toward higher PUFA intake as replacement for SFA would significantly reduce rates of coronary heart disease. In addition, consumption of PUFA has been reported to be associated with a lower incidence of type 2 diabetes (Salmerón et al., 2001). Sphingolipids and their digestion products, such as ceramides and sphingosines, are known to positively affect cell regulation and suppression of intestinal inflammation (Vesper et al., 1999). The effects of supplemental BOV+ on the milk lipidome observed in this study suggests that changes in milk lipid profile as a result of supplementing DFM may offer a healthier profile of FA to consumers.

In conclusion, the results of this study demonstrated that dietary supplementation of a DFM containing a mixture of *L. animalis, P. freudenreichii, B. subtilis*, and *B. licheniformis* to Holstein dairy cows fed an early-lactation diet altered the milk lipidome toward increased relative concentrations of LC-PUFA. This modification of the milk lipid profile offers a healthier profile of FA to consumers with its associated health benefits.
